# JBC reviews tribute to the memory of Dr William (Bill) Smith (1945–2024)

**DOI:** 10.1016/j.jbc.2026.111287

**Published:** 2026-02-12

**Authors:** George M. Carman

This compendium of eight JBC reviews honors the memory of Dr William (Bill) Smith and celebrates his profound scientific contributions and outstanding service to the American Society for Biochemistry and Molecular Biology (ASBMB). As an ASBMB Fellow and an associate editor of the *Journal of Biological Chemistry* (JBC) and *Journal of Lipid Research* (JLR), I had the privilege of nominating Bill to the fellowship of the society and organizing this JBC Reviews tribute.

Bill was a leading researcher in signal transduction, eicosanoids, and lipid mediators. He made significant achievements in underpinning studies on the cyclooxygenase (COX)-2 enzyme, investigating how COX enzymes convert arachidonic acid into prostaglandins, elucidating how these molecules regulate vital processes like blood flow and blood clot formation, and developing nonsteroidal anti-inflammatory drugs. Bill’s scientific stature is exemplified by continuous funding from the National Institutes of Health, his service on national and international advisory boards, and honors and awards he earned.

Beyond his research, Bill was a dedicated mentor to students and fellows. He was also my mentor, editorial colleague, and close friend (Fig. 1). Bill was a pivotal figure in the ASBMB by serving on the Council and as an associate editor of the JBC and a co-editor-in-chief of the JLR. He was elected as an ASBMB Fellow for his exceptional service to the society and distinguished research accomplishments. He received numerous honors, including the Bayer Corporate Senior Aspiring Award, the Michigan State Scientist of the Year Award, The University of Michigan Distinguished Faculty Lectureship Award in Biomedical Research, and ASBMB's Avanti Award in Lipids and William C. Rose Award. Bill is remembered by his colleagues as a brilliant mind with a sharp wit and magnificent sense of humor.

**Figure 1:**
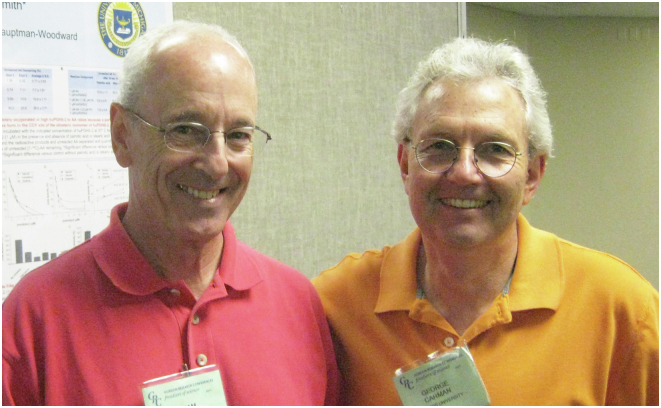
Bill and George at the Gordon Research Conference-Molecular and Cellular Biology of Lipids at Waterville Valley, NH in 2011.

If you have not already, please be sure to read his article in JBC reflections “A seven-step plan for becoming a moderately rich and famous biochemist.” It beautifully narrates Bill’s scientific career, his dedication to mentees and colleagues, and his deep love for his wife Andrea, children, and grandchildren.

This collection of articles contributed by Bill's colleagues (Fig. 2) covers the diverse range of his research interests. Lawrence J. Marnett opens the collection by reviewing the chemistry and biology of endocannabinoid oxygenation by prostaglandin H synthase-2 (COX-2). Next, Michael G. Malkowski discusses the allosteric regulation of prostaglandin endoperoxide H2 synthases. Claus Schneider and Alan R. Brash provide insights into unusual and atypical cyclooxygenase reactions. Hartmut Kühn makes a detailed discussion of the molecular enzymology, evolutionary biology, and biological relevance of lipoxygenase enzymes. Valerie B. O'Donnell offers an update on the biochemistry of bioactive lipids and oxylipins generated through the oxygenation of fatty acids. Ernst H. Oliw provides a comparative discussion, contrasting the catalytic and structural properties of linoleate dioxygenases and their cytochrome P450s with enzymes of the cyclooxygenase cascade. Darryl C. Zeldin reviews the putative receptors and signaling pathways responsible for the biological actions of epoxyeicosatrienoic acids. Ruma Banerjee concludes the collection by discussing the interplay between the chemical power and poison of H_2_S and O_2_ metabolism at the electron transport chain.

**Figure 2:**
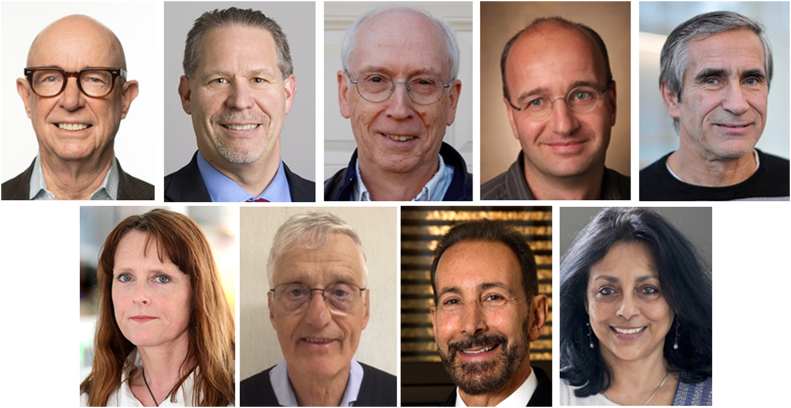
**Corresponding authors of the compendium.***Top row*: Lawrence J. Marnett, Michael G. Malkowski, Alan R. Brash, Claus Schneider, and Hartmut Kühn. *Bottom row*: Valerie B. O'Donnell, Ernst H. Oliw, Darryl C. Zeldin, and Ruma Banerjee.

## Conflict of interest

The author declares that he has no conflicts of interest with the contents of this article.

